# Informal Support Networks of Tanzanians With Chronic Diseases: Predictors of Support Provision and Treatment Adherence

**DOI:** 10.3389/ijph.2022.1605366

**Published:** 2022-11-23

**Authors:** Brady Hooley, Sally Mtenga, Fabrizio Tediosi

**Affiliations:** ^1^ Swiss Tropical and Public Health Institute (Swiss TPH), Basel, Switzerland; ^2^ University of Basel, Basel, Switzerland; ^3^ Ifakara Health Institute, Ifakara, Tanzania

**Keywords:** social capital, chronic conditions, LMICs, network methods, NCDs, Tanzania, social health insurance

## Abstract

**Objectives:** To examine the role of NCD patients’ social ties as informal caregivers and whether receiving their support is associated with engagement in care.

**Methods:** NCD outpatients (N_2_ = 100) in rural Tanzania completed a cross-sectional questionnaire to characterize the support role of their social ties (N_1_ = 304). Bivariate analyses explored predictors of social support and whether social support is associated with engagement in care.

**Results:** This study found that 87% of participants had health insurance, yet 25% received financial support for financing healthcare. Patient gender, age and marital status were found to be important predictors of social support, with NCD-related disability and disease severity being predictive to a lesser degree. Monthly receipt of both material and non-material support were associated with increased odds of adherence to prescribed medications.

**Conclusion:** These findings indicate that patients’ social ties play an important role in filling the gaps in formal social health protection and incur substantial costs by doing so. The instrumental role of even non-material social support in promoting engagement in care deserves greater attention when developing policies for improving this population’s engagement in care.

## Introduction

Non-communicable diseases (NCDs) are becoming more prevalent in sub-Saharan Africa (SSA), now accounting for nearly 50% of all disability-adjusted life-years (DALYs) with cardiovascular diseases, cancers and “other” NCDs being the largest contributors (1–3). This epidemiological transition and double burden of communicable and non-communicable diseases in SSA has made it difficult for health systems to adapt to the challenge of financing and delivering chronic care. In fact, health systems in these countries still mainly focus on providing acute episodic treatment and remain under-equipped for providing long-term treatment for NCDs (4, 5). The lack and inequitable allocation of funding for NCDs has exacerbated human resource challenges, and contributed to the poor availability of NCD diagnosis and care services outside major towns and urban centres (6). This has contributed to poorer health outcomes for Africans with NCDs, with age-standardised mortality attributable to NCDs being up to three times higher among SSA populations than European ones (6, 7).

While the Global Burden of Disease study has indicated that Universal Health Coverage has been improving in general (8), population coverage by social health protection schemes in SSA remains low and tends to favour wealthier population groups (9, 10). Rather, direct out-of-pocket payments (OOPs) represent up to 40% of overall health expenditure in LMICs (11–13), creating a significant barrier to accessing healthcare and imposing a disproportionately high financial burden upon vulnerable populations (11, 14). Regardless of whether one subscribes to a social health protection scheme, beneficiaries living outside major towns or urban centres may still face inequities in financial risk protection as a result of high transportation time and costs, and the poor availability of necessary medicines at accredited non-urban health facilities and pharmacies (15, 16).

In addition to OOPs, a substantial burden of indirect costs attributable to NCDs has also been documented (17, 18). A recent systematic review reported that the indirect costs of diabetes can be 1.3–2.1 times higher than the direct medical costs when accounting for patients’ lost productivity, disability and transport-related expenditures (18). A study on the financial burden of diabetes on patients in Mali found substantial opportunity and indirect costs associated with their condition and that they incur higher indirect costs relative to a general sample of individuals seeking healthcare (17). This study also reported that in order to finance the costs of care, patients with diabetes tend to borrow money from their family and friends more frequently than patients without diabetes (17).

While people living with NCDs may enrol in social health protection in order to cope with the direct financial cost of accessing healthcare, patients must still deal with the indirect costs of accessing care and with reduced abilities to work or perform activities of daily living (19, 20). When patients face a substantial burden of opportunity costs or when formal social health protection schemes fail to adequately protect against catastrophic OOPs, many turn to their social network for support (21, 22).

Patients’ social capital may then function as a form of informal social protection, by which social ties provide either material or non-material support to overcome barriers to accessing and adhering to NCD care, and to alleviate other challenges associated with aging or living with a chronic disease (20, 23). Previous research on personal support networks of older adults reports that this population is more likely to have smaller networks with stronger social ties than younger individuals (23–25). Older adults with greater care needs also tended to have a personal network composed mostly of first-degree relatives, partially explaining the higher prevalence of strong ties within this population (23–25).

There is a lack of research from Tanzania and sub-Saharan Africa that investigates patients’ social support networks and whether social support is associated with engagement in care (23, 26). With the exception of recent work in South Africa (23, 27), most literature from sub-Saharan Africa does not employ true egocentric network sampling and methodologies or if they do, they focus on other topics such as HIV transmission or business networks (28–31).

To fill this gap, this study used an egocentric network methodology to characterise patients’ personal networks and measure the material and non-material support that patients receive from their networks. We also sought to explore predictors of social support and whether the amount and type of social support are associated with patients’ retention in NCD care.

## Methods

This study used a cross-sectional personal network survey to examine the composition and structure of NCD patients’ informal support networks, and the function of informal social support as it relates to coping with chronic disease. Similar in concept to the more common sociocentric social network analysis, the egocentric methodology we employed asked participants to provide information on themselves (the “ego”) and on the people in their social environment (referred to as “alters”). This approach allowed us to analyse the composition and function of a network of actors in relation to individuals living with a chronic condition (27, 32, 33).

### Setting and Sample

This study recruited 100 patients with at least one previously diagnosed NCD. We recruited them following their visit to outpatient clinics at the St. Francis Referral Hospital and Kibaoni Health Centre; both located in Ifakara, the principal town in the rural Kilombero District of Tanzania. The St. Francis Referral Hospital serves as the referral hospital for all of Kilombero district, while the Kibaoni Health Centre is a large secondary health facility north of Ifakara. Patients seeking care for NCDs are routinely unable to receive appropriate care at primary care facilities (34), so a health centre and a hospital were purposively selected to facilitate the recruitment of our target population.

In July and August 2021, research assistants recruited potential respondents in the outpatient waiting room and administered the questionnaire in Kiswahili after their consultation. The questionnaire was accompanied by blood pressure and random blood glucose measurements. Inclusion criteria were that participants be at least 18 years of age and have a chronic health condition that was previously diagnosed by a healthcare provider. Research assistants provided examples of chronic conditions that included both NCDs such as diabetes and disabilities such as paralysis so that patients with a wide range of informal support needs could be recruited. Patients were excluded if they showed signs of cognitive impairment. Data was collected using tablets and Open Data Kit (35), and data was uploaded to a secure server hosted by the Ifakara Health Institute in Tanzania at the end of each day of data collection. In order to maintain data privacy, this server is accessible only to the Ifakara Health Institute data manager and to the authors of this manuscript.

### Questionnaire

The first stage of the questionnaire asked participants (egos) for information on basic sociodemographics, health insurance enrolment, and their chronic diseases. The second stage used a “name generator” to ask participants to (32, 33, 36):

‘Please list up to 10 people in your social environment. List people that you have talked to either in person or by phone or internet, at least one time in the past 6 months. This list can include people such as your family members, friends, neighbours, or elders. Please start by listing the 5 people (adults) who are the most important to you for any reason, and indicate the type of relationship that you have with this person’.

The third stage of the questionnaire asked participants a loop of questions for each named social tie (alter) elicited by the name generator. These questions asked about the attributes of each alter, such as their age, gender and residential proximity to the ego.

We then asked egos about the frequency of contact (in-person, by phone or otherwise) with each alter and the support that the alter provides. We began by generically asking participants, “has [alter] supported you in coping with your chronic disease in the past 6 months? “Support” may include emotional support, prayer, food, time, labour, money or sharing helpful knowledge and information.” Subsequent questions then gathered more information on the following three sub-types of support (37, 38):

- Emotional support, such as providing comfort to the ego, making them feel respected or loved, or praying with/for them
- Informational support, such as sharing advice and knowledge, or helping to understand doctor’s instructions
- Material support, such as giving money for healthcare or bus fare, providing transport to the health facility, or cooking and helping with other tasks at home

As we were mostly interested in learning if the determinants of and outcomes of receiving material support were different from intangible forms of support in general, emotional support and informational support were pooled together as non-material support for analysis.

Categorical responses to the frequency of social contact and social support were converted to a count of days of contact or support provided by an alter each month, such that “monthly” communication or support events were valued as 1, “a few times a month” as 2, “weekly” as 4, “a few times a week” as 10, and “daily/almost daily” as 30 days per month (23). This method of approximating these categorical responses as a count of days of support or contact provided per month better allowed these variables to be summarized at the ego level as person-days of contact or support received per month and “adjusts for the unequal gaps between frequency categories as collected” (23).

For alters who provided material support, we asked more detailed information about the type of material support and the amount of support given in the case that an alter gave money to an ego.

Lastly, participants were asked to answer to the best of their knowledge whether two alters knew one another, which was used to describe and visualize network structure. While there are methods of eliciting more detailed information about alter-alter ties, we chose this simple method in order to minimize recall bias and participant fatigue (39, 40).

### Analysis

Descriptive statistics explored the composition and function of participants’ support networks while bivariate analyses identified potential predictors of support provision and predictors of egos’ adherence to NCD treatment. We used bivariate analyses to identify potential predictors of social support, measured as the number of days of support over the past month. For variables at the alter level we used linear regression while adjusting for clustering at the ego level and for variables at the ego level we used unequal variance t-tests.

To investigate the association between social support and adherence to NCD treatments, non-material and material support were aggregated at the ego level and support was dichotomized by whether or not the participant received non-material or material support over the past month. We then calculated unadjusted risk ratios and unadjusted Cornfield’s odds ratios for the association between receiving social support at least monthly and adherence to medications. Due to the relatively small sample size, we had insufficient degrees of freedom for computing adjusted odds ratios. We used Python 3.9.7 and the “NetworkX” packgage (41) for network visualisation and STATA version 16 for analyses (42).

### Variables of Interest

#### Outcomes

At the level of ego-alter ties, the main outcomes of interest were the frequency of non-material and material social support measured as the number of instances of support over the last month. At the ego level we were interested in adherence to NCD medication. Adherence was a binary variable for whether or not the patient had taken their prescribed NCD medications within the past 7 days.

We also aggregated several tie-level variables to create a composite measure of relationship strength, ranging from zero (no relationship) to one (the strongest possible relationship). This aggregate measure included variables for alters’ residential proximity to the ego, duration of relationship, frequency of communication or contact, frequency of social support provided by the alter, reciprocity of support by ego, provision of non-material support, provision of material support and egos’ satisfaction with alters support. Non-binary categorical variables were first scaled to a value between zero and one before being averaged with the other included variables to produce the relationship strength scores. We used this aggregate measure of tie weight to facilitate the visualization of individual ego networks.

#### Predictors

The main ego-level predictor variables of interest were age, sex, marital status, multimorbidities, health insurance status and whether one’s NCD affects their ability to work. At the alter level, we included sex, age, relationship to ego, and interactions between alter age and sex with ego age and sex. The rationale for investigating potential interaction effects between ego and alter gender stems from evidence suggesting that there are gender inequities in both the provision and receipt of social support (43, 44). The interaction between ego and alter age was included because past research on personal networks has indicated that people tend to associate with those similar to them (44, 45). In this case, where we investigate an older population with NCDs, a tendency for participants to report more social ties with individuals similar to themselves would be detrimental to the formation of social capital that could assist them coping with their NCD(s) (25). For the purposes of this analysis, alter age was dichotomized for alters who are younger than the ego and for alters who are the same age or older than the ego.

In investigating participant medication adherence, we also used predictors at the ego-alter tie level. We were mainly interested in determining if at least monthly provision of social support would predict medication adherence. We also present bivariate analyses demonstrating the association between health insurance status and medication adherence as a suspected confounder.

### Ethics Statement

This study received ethical approval from the Ifakara Health Institute Institutional Review Board (Ref: IHI/IRB/AMM/No: 13- 2021) and the Tanzanian National Institute for Medical Research (Ref: NIMR/HQ/R.8a/Vol. IX/3518). Prior to participants’ recruitment, we presented the study’s objectives and explained that participants would be asked to provide information on their own demographics and health status and relationship details of members of their social network. All potential participants provided written informed consent prior to participation, and were informed that they may refuse biometric measurements and/or withdraw from the study at any time without consequences. In cases where potential participants were unable to write, we accepted verbal consent in lieu of written consent.

## Results

### Ego Characteristics

The 100 participants provided information on relationship characteristics and social support provision for 304 social ties. Egos had a mean age of 63 years and 68% were women. Most egos had only primary education (56%) and listed their primary occupation as subsistence farming (68%), with only 16% having reported doing paid work within the last year (Table 1).

**TABLE 1:** Summary of key ego-level variables with chi-square *p*-values, disaggregated by ego gender. An excerpt of individual patient networks is provided in Supplementary Figure S1 (Ifakara, Tanzania, 2021).

		Overall	Women	Men	*p*-value
n		100	68	32	
Age, mean (SD)		62.8 (8.2)	61.1 (7.8)	66.4 (8.0)	0.002**
Marital status (%)	Married	57.0	41.2	90.6	0.001**
	Widowed	39.0	52.9	9.4	
	Divorced	1.0	1.5	0.0	
	Living with partner	1.0	1.5	0.0	
	Never Married	1.0	1.5	0.0	
	Separated	1.0	1.5	0.0	
Education (%)	None	1.0	1.5	0.0	0.087
	Primary	56.0	58.8	50.0	
	Some primary	12.0	16.2	3.1	
	Some secondary	4.0	4.4	3.1	
	Secondary	21.0	16.2)	31.2	
	College	6.0	2.9	12.5	
Household size, mean (SD)		4.7 (2.2)	4.9 (2.4)	4.2 (1.8)	0.113
Occupation (%)	Subsistence Farmer	68.0	64.7	75.0	0.087
	Self-employed, small business	10.0	13.2	3.1	
	Public Servant	7.0	8.8	3.1	
	Retired	7.0	4.4	12.5	
	Caring for home/children	5.0	7.4	0.0	
	Private Formal Sector	3.0	1.5	6.2	
Paid work in last 6 months (%)	Yes	16.0	14.7	18.8	0.824
Does chronic condition ever prevent you from working? (%)	Never	59.0	57.4	62.5	0.105
	Sometimes	37.0	41.2	28.1	
	Completely	4.0	1.5	9.4	
Days in last month that chronic condition has prevented work, mean (SD)		10.2 (8.7)	9.3 (7.5)	12.5 (11.2)	0.380
Current health insurance (%)	Yes	87.0	85.3	90.6	0.541
Type of health insurance (%)	iCHF^a^	12.6	10.3	17.2	0.525
	NHIF^b^	86.2	87.9	82.8	
	Other	1.2	1.7		
Did participant pay for own health insurance? (%)	I do not know	1.1	1.7		0.014*
	No	44.8	55.2	24.1	
	Yes, partially	2.3	3.4		
	Yes, completely	51.7	39.7	75.9	
Current fee exemption (%)	Yes	14.0	14.7	12.5	1.000
Perceived health status (%)	Bad	8.0	11.8	0.0	0.184
	Moderate	26.0	23.5	31.2	
	Good	65.0	63.2	68.8	
	Very good	1.0	1.5	0.0	
Diabetes (%)	Yes	24.0	23.5	25.0	1.000
Hypertension (%)	Yes	92.0	94.1	87.5	0.264
Epilepsy (%)	Yes	1.0	0.0	3.1	0.320
Asthma (%)	Yes	2.0	1.5	3.1	0.540
Other chronic condition, (%)	Yes	12.0	11.8	12.5	1.000
Systolic blood pressure, mean (SD)		143.2 (18.5)	140.8 (18.5)	148.3 (17.7)	0.055
Diastolic blood pressure, mean (SD)		92.8 (13.5)	91.0 (13.6)	96.6 (12.7)	0.048*
Stage II Hypertension (%)	Yes	63.0	58.8	71.9	0.299
Random blood glucose, mean (SD)		6.1 (2.7)	6.1 (2.6)	6.1 (2.8)	0.989
Number of named alters, mean (SD)		3.0 (1.0)	3.0 (1.1)	3.2 (0.7)	0.357

^a^ Improved Community Health Fund.

^b^ National Health insurance Fund.

*p < 0.05, **p < 0.01, ***p < 0.001.

All participants had at least one chronic condition at enrolment, with 29% having two or more. Hypertension was the most commonly reported NCD (92%), followed by diabetes (24%), asthma (2%) and epilepsy (1%). Most participants (66%) reported their health status as being “good” or “very good.” All participants reported having received formal care for their NCD(s) and 85% reported having taken medication for their condition within the past 7 days.

Egos named an average of three alters, ranging from two to ten alters. First-degree relatives accounted for 84% (IQR: 67%–100%) of participants’ alters, followed by other family members (11%, IQR: 0%–25%), and other ties (4.65%, SD: 16.6). Egos’ networks were small and dense, in that alters were highly interconnected with only two egos naming an alter that did not know all of the ego’s other alters.

Egos’ mean tie weight was 0.77 (SD: 0.07). When using k-means clustering to divide tie weight into a three-level ordinal variable, 12% of participants had weak ties on average, followed by 39% with medium-weight ties and 49% with strong ties (See Supplementary Figure S1 for excerpt of individual sociograms).

Of all egos, 86% reported receiving emotional support, 74% received informational support, while only 43% received material support from their network over the 6-month recall period. Of those who received material support, 62% reported receiving money, with a total of Tsh 97,620 ($42USD) on average (SD: 100,141; IQR: 15,000–140 000).

### Alter Level

Egos’ children were the most frequently named alter relation (64%), followed by a significant other (17%), other family members (12%) and “other” alters (6.6%). Alters mostly lived in the same household or village as the egos (56% and 17% respectively) and tended to be younger than the egos themselves, with 57% of alters being younger than the participants (Table 2).

**TABLE 2:** Summary of key alter variables with chi-square *p*-values, disaggregated by alter gender (Ifakara, Tanzania, 2021).

		Overall	Women	Men	*p*-value
n		304	136	168	
Alters relationship to ego (%)	A child	64.1	58.1	69.0	0.004**
	Significant other	17.1	18.4	16.1	
	A parent	1.3	2.9	0.0	
	Friend	0.7	1.5	0.0	
	Neighbour	5.6	2.9	7.7	
	Other	0.3	0.0	0.6	
	Other family member	10.9	16.2	6.5	
Homophily on gender (%)	Yes	43.8	61.8	29.2	<0.001***
Alter age (%)	Less than 20 years old	1.0	1.5	0.6	<0.001***
	20–30 years old	28.3	25.7	30.4	
	30–40 years old	28.0	17.6	36.3	
	40–50 years old	20.1	28.7	13.1	
	50–60 years old	13.8	17.6	10.7	
	More than 60 years old	8.9	8.8	8.9	
Homophily on age (%)	Yes	9.5	10.3	8.9	0.836
Tie weight, mean (SD)		0.8 (0.1)	0.8 (0.1)	0.8 (0.1)	0.285
Time known (%)	Less than 1 year	2.3	1.5	3.0	0.603
	1–5 years	19.4	22.1	17.3	
	5–10 years	9.9	10.3	9.5	
	More than 10 years	68.4	66.2	70.2	
Proximity (%)	In the same household	55.9	55.1	56.5	0.377
	In the same village/town	16.8	14.7	18.5	
	In the same district	5.3	8.1	3.0	
	In another district in Morogoro	3.9	4.4	3.6	
	In another region	17.8	17.6	17.9	
	In another country	0.3	0.0	0.6	
Frequency of contact (%)	Every day	70.1	72.1	68.5	0.366
	A few times a week	15.5	13.2	17.3	
	Once a week	11.8	13.2	10.7	
	A few times a month	1.6	0.7	2.4	
	Once a month	0.7	0.0	1.2	
	Less than once a month	0.3	0.7	0.0	
Ego’s satisfaction with support (%)	Neutral	1.3	0.0	2.4	0.177
	Satisfied	33.6	35.3	32.1	
	Very satisfied	65.1	64.7	65.5	
Frequency of support over past 12 months, (%)	It has decreased	1.6	0.0	3.0	0.124
	It has not changed	90.8	91.9	89.9	
	It has increased	7.6	8.1	7.1	
Does alter provide emotional support? (%)	Yes	84.9	87.5	82.7	0.322
Frequency of emotional support (%)	Every day	1.2	0.8	1.4	0.040*
	A few times a week	0.8	0.0	1.4	
	Once a week	0.8	0.0	1.4	
	A few times a month	14.3	20.2	9.3	
	Once a month	29.7	32.8	27.1	
	Less than once a month	53.3	46.2	59.3	
Does alter provide informational support? (%)	Yes	73.4	82.4	66.1	0.002**
Frequency of informational support (%)	A few times a week	0.9	0.0	1.8	0.010*
	Once a week	4.9	5.4	4.5	
	A few times a month	14.7	22.3	7.1	
	Once a month	36.2	35.7	36.6	
	Less than once a month	43.3	36.6	50.0	
Does alter provide material support? (%)	Yes	41.1	37.5	44.0	0.300
Does alter provide both material support and a form of non-material support? (%)	Yes	32.2	32.4	32.1	0.969
Frequency of material support (%)	Every day	5.6	5.9	5.3	0.080
	A few times a week	26.2	25.5	26.7	
	Once a week	9.5	17.6	4.0	
	A few times a month	21.4	25.5	18.7	
	Once a month	11.9	7.8	14.7	
	Less than once a month	25.4	17.6	30.7	
Type of material support: money (%)	Yes	94.4	96.1	93.3	0.700
Type of material support: transport (%)	Yes	33.6	39.2	29.7	0.362
Type of material support: other (%)	Yes	6.4	9.8	4.1	0.269
Amount of money provided (TSH), mean (SD)		50861.3 (64163.1)	56285.7 (74625.2)	47064.3 (55954.9)	0.466
Purpose of money: clinic/pharmacy fees (%)	Yes	25.4	33.3	20.0	0.139
Purpose of money: transport fare (%)	Yes	49.6	49.0	50.0	0.941
Purpose of money: other (%)	Yes	68.3	78.4	61.3	0.067

*p < 0.05, **p < 0.01, ***p < 0.001.

The support provided by alters was typically satisfactory to egos, and the frequency of support during the past 6 months was stable (Table 2). At the alter level, 85% of ties provided emotional support, 73% provided informational support and 41% provided material support. However, when filtering supportive ties based on whether alters provided support at least monthly, it was found that only 39% of alters provided monthly emotional support, 41% provided monthly informational support and 31% provided monthly material support.

Relative to emotional and informational support, alters were less likely to provide material support. Yet, those that did so provided material support more frequently than alters whose primary role was to provide emotional or informational support. Of the 41% of alters that provided material support, 75% provided it at least monthly while 26% provided material support several times a week.

Money was the most frequently reported form of material support (94%), followed by providing transport (34%) and services or goods such as helping to care for the home or cooking (6.4%). The last time an alter provided money to the ego, they gave a mean of Tsh 50,861.3 ($22USD) (SD: 64,163.1). Of those that provided money, 25% did so for paying medical fees, 50% for the purpose of paying transport fare, and 68% for food or other household goods.

### Predictors of Social Support and Treatment Adherence

**FIGURE 1:**
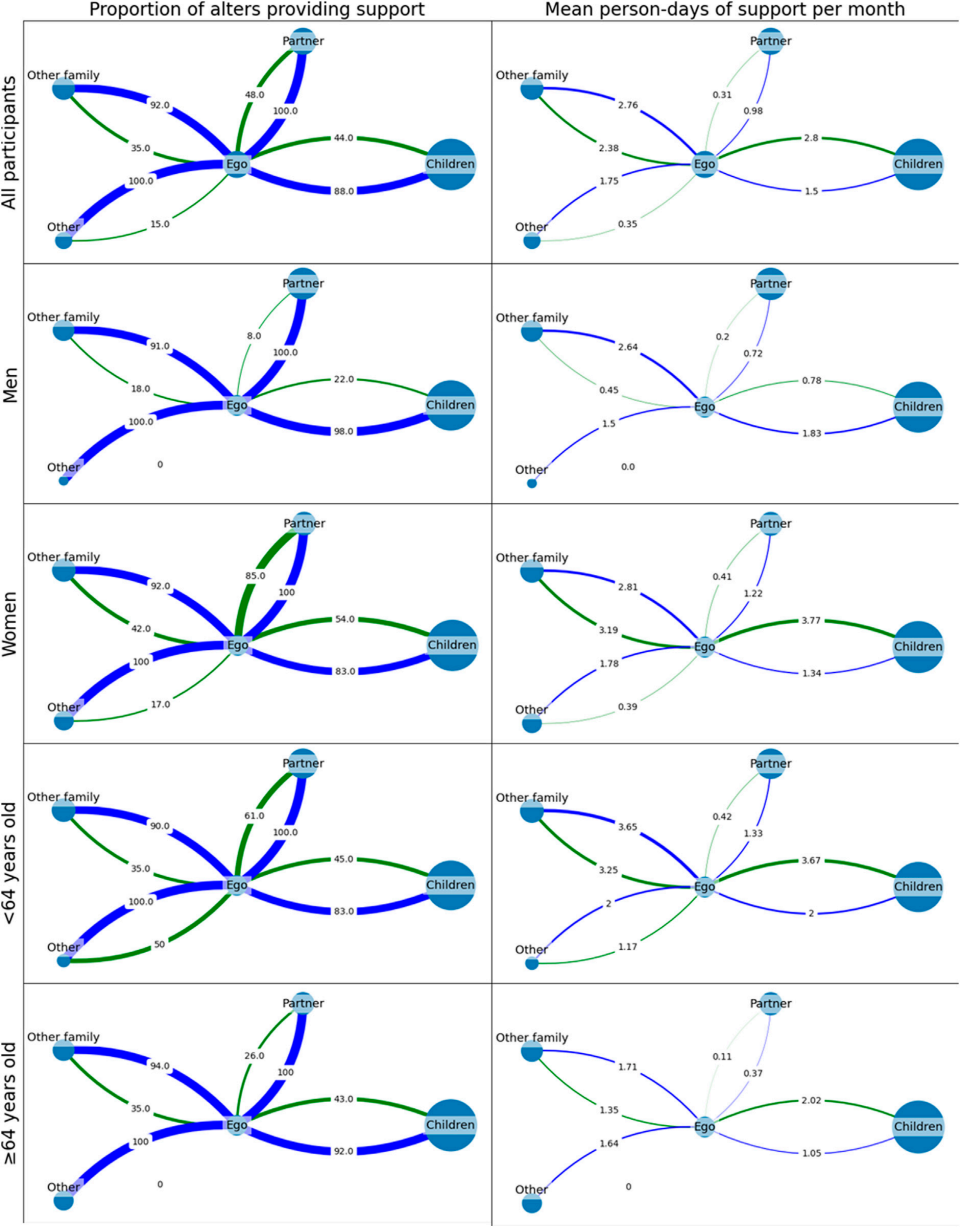
Average cluster graphs depicting the amount of support received by participants from their alters. Alter node size represents the relative prevalence of each relation type within participants’ personal support networks. The left column represents the proportion of alters that provide non-material support (blue) and material support (green). The right column represents the average number of support events provided by each type of alter per month (Ifakara, Tanzania, 2021).

Men had more instances of social contact per month than women (Supplementary Table S1); however, this did not translate to them receiving more social support per month (Table 3). There was no difference between men and women in terms of the receipt of non-material support, while women received significantly more material support than men (Figure 1; Table 3). Participants older than 64 years of age experienced as much social contact with their alters per month as did younger participants (Supplementary Table S1). However, they received significantly less non-material and material support than their younger counterparts (Table 3). Participants who were widowed or otherwise not living with a significant other experienced on average 12 fewer instances of social contact per month than those living with a significant other (Supplementary Table S1), yet they received significantly more material support (Figure 1; Table 3).

**TABLE 3:** Mean differences in number of social support events between levels of ego-level and alter-level predictor variables. Significance testing used unequal variances t-tests for predictor variables at the ego level, and linear regressions adjusted for ego-level clustering for predictor variables at the alter level (Ifakara, Tanzania, 2021).

Variable	Variable level	Non-material support events per month			Material support events per month		
		Mean (SE)	95% CI	p-value	Mean (SE)	95% CI	*p*-value
Ego gender	Women (*n* = 68)	4.6 (0.81)	3.01–6.25	0.6391	8.8 (1.4)	5.97–11.62	<0.0001***
	Men (*n* = 32)	5.2 (1.2)	2.67–7.64		1.8 (0.75)	0.32–3.37	
Ego age	<64 years (*n* = 51)	6.1 (1.2)	3.77–8.51	0.0202*	8.3 (1.7)	4.96–11.66	0.0428*
	>64 years (*n* = 49)	3.4 (0.56)	2.27–4.54		4.8 (1.2)	2.37–7.15	
Marital status	Widowed (*n* = 42)	5.7 (1.2)	3.24–8.18	0.1374	8.8 (2.0)	4.74–12.9	0.0459*
	Living with a partner (*n* = 58)	4.1 (0.75)	2.64–5.64		4.9 (1.0)	2.93–6.97	
NCD affects ability to work	No (*n* = 59)	3.7 (0.57)	2.59–4.87	0.0446*	6.1 (1.4)	3.30–8.94	0.2999
	Yes (*n* = 41)	6.3 (1.40)	3.52–9.17		7.2 (1.6)	4.09–10.35	
Multimorbidities	No (*n* = 72)	4.2 (0.74)	2.70–5.66	0.0889	4.9 (1.1)	2.72–7.05	0.0115*
	Yes (*n* = 28)	6.4 (1.43)	3.45–9.33		10.9 (2.3)	6.19–15.60	
Stage II Hypertension	No (*n* = 37)	6.9 (1.4)	4.12–9.72	0.0161*	11.0 (2.0)	6.86–15.08	0.0016**
	Yes (*n* = 63)	3.6 (0.65)	2.25–4.86		4.0 (1.0)	1.93–6.04	
**Alter-level variables**		**Mean difference (SE)**	**95% CI**	***p*-value**	**Mean difference (SE)**	**95% CI**	**p-value**
Alter gender, relative to women (*n* = 136)	Men (*n* = 168)	−0.22 (0.39)	−0.99–0.57	0.581	0.11 (0.57)	−1.03–1.24	0.855
Alter gender x ego gender interaction, relative to woman x woman (*n* = 84)	Woman x man (*n* = 52)	−0.41 (0.47)	−1.35–0.52	0.379	−2.90 (0.72)	−4.34–1.48	<0.001***
	Man x woman (*n* = 119)	−0.52 (0.49)	−1.50–0.46	0.293	−0.46 (0.84)	−2.13–1.21	0.587
	Man x man (*n* = 49)	−0.020 (0.76)	−1.54–1.50	0.979	−2.34 (0.84)	−4.00–0.67	0.006**
Alter age, relative to alters younger than ego (*n* = 271)	Same age or older than ego (*n* = 33)	−0.34 (0.39)	−1.12–0.44	0.385	−1.64 (0.52)	−2.68–0.60	0.002**
Daily contact with alter, relative to less than daily contact (*n* = 91)	Daily contact (*n* = 213)	−2.14 (0.62)	−3.37–0.91	0.001**	−3.50 (0.92)	−5.33–1.68	<0.001***
Relationship type, relative to children of alters (n = 195)	Partner (*n* = 52)	−0.52 (0.35)	−1.21–0.18	0.143	−2.49 (0.50)	−3.48–1.51	<0.001***
	Other family (*n* = 37)	1.26 (0.89)	−0.51–3.03	0.162	−0.42 (1.03)	−2.47–1.63	0.684
	Other (*n* = 20)	0.25 (0.57)	−0.88–1.38	0.658	−2.45 (0.54)	−3.52–1.38	<0.001***
Alter residence, relative to residing in a different household (*n* = 134)	Within household (*n* = 170)	−1.61 (0.43)	−2.47–0.75	<0.001***	−4.42 (0.73)	−5.89–2.97	<0.001***

Support predictors at the alter level were largely not associated with the provision of non-material support (Table 3). However, almost all alter-level predictors were associated with the provision of material support (Table 3).

Unadjusted risk ratios and odds ratios indicated that the receipt of both non-material support and material support in the past month are associated with the patient having taken their medicine in the last week (Table 4).

**TABLE 4:** Two-way tables showing the association between monthly non-material and material support and whether a participant took their medicines as prescribed within the past 7 days (Panel A: Unadjusted risk ratio: 1.57 (95% confidence interval: 1.20–2.05), unadjusted Cornfield’s odds ratio: 19.80 (95% confidence interval: 4.54-.), *p* < 0.0001; Panel B: Unadjusted risk ratio: 1.38 (95% confidence interval: 1.15–1.67), unadjusted Cornfield’s odds ratio: 21.00 (95% CI: 3.33-.), *p* = 0.0001) (Ifakara, Tanzania, 2021).

	Adherent (%)	Non-adherent (%)	Total (%)
Panel A: Non-material support			
Monthly	64	2	66
Less than monthly	21	14	34
Total	85	15	100
Panel B: Material support			
Monthly	51	1	52
Less than monthly	34	14	48
Total	85	15	100

## Discussion

This exploratory study contributes to the small but growing use of egocentrically sampled network data in sub-Saharan Africa and provides a first look at personal networks and social support for patients with NCDs in Tanzania. The findings indicate age, gender and marital status are associated with differences in the amount of social support that one receives from their social network and that receiving support was associated with higher odds of treatment adherence.

Participants in this study reported having small, dense social support networks. While this study did not include a younger comparison population, previous research from South Africa and Brazil indicates that older adults and those with greater care needs have smaller network sizes and higher proportions of family ties relative to younger adults (23, 25). Similar to other studies conducted in South Africa, older individuals had fewer social contacts and more “close” or “strong” ties in their network, yet received less support from their networks than younger participants (23, 27). If we compare our population’s experience with NCDs to the experience of people with cognitive decline (27), these findings support the convoy model in that only the closest, most familiar ties are maintained as a patients’ needs for informal care increases (27, 46).

Having a high proportion of strong ties and family ties in a personal network can also be detrimental to forming and mobilising social capital. While strong family ties play a significant role in providing material support (24), a study of low-income households in Brazil indicated that those with closed networks composed primarily of close relatives tend to be isolated from other households or social units in the community (25). On the other hand, networks that incorporate a larger proportion of weak ties from more diverse backgrounds may mobilise a larger total amount of social capital (21, 25). To make an analogy between personal support networks and social health protection schemes, the presence of more diverse ties within one’s social network would be comparable to increasing a scheme’s risk pool and reducing fragmentation (21, 47). This may in part explain why women receive more social support than men, as their networks appear to contain more diverse non-kin social ties and they receive support from a more diverse range of social contacts compared to men (Figure 1) (48–50).

Consistent with the findings of other studies (30, 37), we found that higher levels of non-material and material support are associated with better adherence to care. While this finding may support the hypothesis that social networks and social support are instrumental in the promotion of well-being and healthy behaviours, the employed methodology cannot establish a causal relationship (30, 37). In addition to networks’ role in providing financial support to assist patients in overcoming financial barriers to healthcare, there is evidence to suggest that non-material social support can also influence patients’ health outcomes by promoting healthy behaviours, such as adherence to treatments and remaining engaged in care (37, 51).

While most of the study participants were covered by health insurance, most were included on a family member’s insurance plan or did not pay for their own insurance. Furthermore, many participants received additional financial support to assist with both direct and indirect costs of care. These findings and those of previous work support the importance of health insurance schemes allowing the inclusion of secondary beneficiaries as dependents, as patients often try to capitalise on their social networks by being added as beneficiaries on family members’ health insurance memberships (52).

The provision of material support to insured patients can be further explained by the inclusions and exclusions of social health protection schemes. As access to private pharmacies and tertiary health facilities is often excluded or restricted from benefits packages in Tanzania, beneficiaries whose medications are not routinely stocked by public pharmacies or lower-level health facilities may incur higher indirect costs and OOPs by self-referring to higher level facilities (53–55). Informal social support fills this gap in social health protection, indicating that health insurance does not adequately protect people with NCDs from the economic consequences of their disease. Interventions and efforts to scale up and improve the utility of health insurance should thus consider the social support structure of beneficiaries and patients and that the needs of patients with NCDs often cannot be met by existing benefits packages (16, 56). Furthermore, the provision of even non-material support represents an opportunity cost for informal caregivers whereby key caregivers may experience a high burden of support responsibilities with implications upon their own health and socioeconomic conditions (44).

### Limitations and Policy Implications

These findings should be interpreted bearing in mind several study limitations. The small sample size did not allow us to build models that control for confounding, while the study and questionnaire design did not allow us to establish temporal precedence regarding whether receiving social support leads to improved wellbeing, if healthier patients were already in a relatively good state of health due to the buffering effect of a supportive network, or if healthier patients simply do not require as much support from their network. Future studies should aim to empirically study the association between social support and patients’ well-being and health outcomes. In addition, the use of a name generator to prompt the naming of alters tends to bias the naming of stronger social ties than other methods (57, 58). While we can assume that our sampling method elicited participants’ strongest ties who would be most likely to provide informal care and support, we do not know how the number of caregivers in a network compares to the overall network size.

Lastly, this study focused only on patients seeking healthcare services within the rural town of Ifakara, so these findings may not generalize to the broader Tanzanian population. Nevertheless, while this study provided interesting insights regarding the role of social support in a low-income rural setting, we could strengthen these findings in the future by comparing this rural sample with one from an urban setting.

Despite the limitations of this study, the results indicate some important policy implications. Policy makers should pay attention not only to this patient group, but also keep in mind patients’ social networks who, for the time being, at least partially absorb the cost of patients’ unmet material and non-material support needs. Furthermore, as the population of SSA continues to age, the ability of younger generations to informally support their elders may diminish and there is evidence that evolving social norms and urbanisation have already begun to erode the reliability with which elders receive informal support (49, 59).

In order to both relieve caregivers of this burden and ensure that the elderly and those living with chronic diseases can age with dignity and lead fulfilling lives, there are several measures that policymakers could consider, such as: scaling up health insurance coverage to reduce the burden of out-of-pocket healthcare expenditures by the elderly and their informal caregivers; and, promoting the decentralization of care for common cardiovascular and metabolic NCDs in order to mitigate the burden of direct, non-medical costs incurred by patients when they have to reach hospitals.

### Conclusion

The informal support networks of NCD patients living in rural Tanzania play an instrumental role in facilitating access to care and filling gaps left by social health protection schemes. People who receive either monthly non-material or material support have significantly higher odds of being adherent to their prescribed medicines. These findings indicate that even though the majority of this patient group is registered with a health insurance scheme, patients continue to receive financial contributions from their social network for the purpose of facilitating their access to healthcare services. It is therefore important to improve the decentralisation of chronic care services and to promote social protection programs that more comprehensively support people with chronic conditions and their support networks in coping with the social and economic consequences of their disease.
