# Supplementary material for: Informal Support Networks of Tanzanians With Chronic Diseases: Predictors of Support Provision and Treatment Adherence
Source: Int J Public Health. 2022 Nov 23;67:1605366. doi: 10.3389/ijph.2022.1605366 (PMC9726723; doi:10.3389/ijph.2022.1605366)
Supplement: Supplementary file 1 [file DataSheet1.DOCX]

Informal support networks of Tanzanians with chronic diseases: Predictors of support provision and treatment adherence

International Journal of Public Health


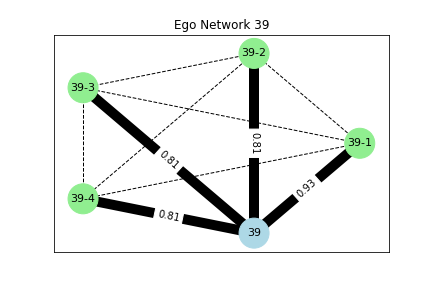

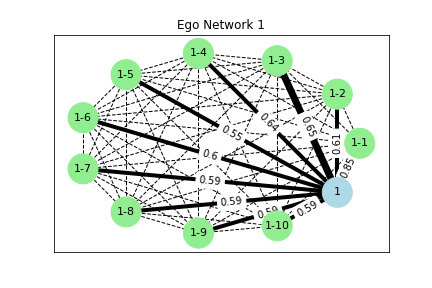

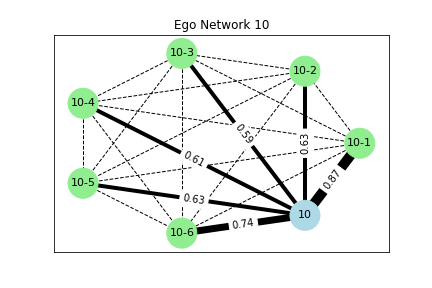

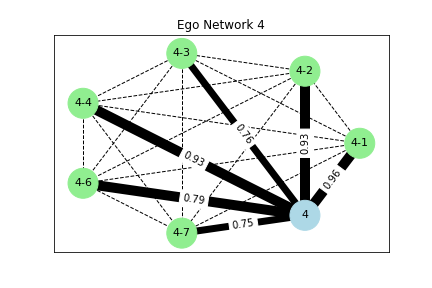


Figure S1: Excerpt of individual-level ego networks. Egos represented in blue, named alters in green, and tie strength represented by line thickness with the line label describing tie strength on a scale from 0-1. Dotted lines represent alter-alter ties (Ifakara, Tanzania, 2021).

Table S1: Mean differences in number of monthly social contact events between levels of ego-level and alter-level predictor variables. Significance testing used unequal variances t-tests for predictor variables at the ego level, and linear regressions adjusted for ego-level clustering for predictor variables at the alter level (Ifakara, Tanzania, 2021).

| Variable | Variable level | Social contact events per month | | |
| --- | --- | --- | --- | --- |
|  |  | Mean (SE) | 95% CI | p-value |
| Ego gender | *Women (n=68)* | 66.6 (3.39) | 59.83-73.35 | 0.037 |
|  | *Men (n=32)* | 77.75 (5.22) | 67.32-88.18 |  |
| Ego age | *< 64 years (n=51)* | 67.41 (3.71) | 59.95-74.87 | 0.165 |
|  | *> 64 years (n=49)* | 73.02 (4.37) | 64.23-81.81 |  |
| Marital status | *Widowed (n=42)* | 63.71 (4.42) | 54.79-72.64 | 0.028 |
|  | *Living with a partner (n=58)* | 74.83 (3.66) | 67.50-82.16 |  |
| NCD affects ability to work | *No (n=59)* | 74.53 (3.88) | 66.75-82.30 | 0.030 |
|  | *Yes (n=41)* | 63.88 (4.03) | 55.74-72.02 |  |
| Multimorbidities | *No (n=72)* | 73.81 (3.23) | 67.37-80.24 | 0.026 |
|  | *Yes (n=28)* | 60.79 (5.67) | 49.16-72.41 |  |
| Stage II hypertension | *No (n=37)* | 67.46 (4.19) | 58.97-75.95 | 0.226 |
|  | *Yes (n=63)* | 71.75 (3.83) | 64.10-70.39 |  |
| Alter-level variables | | Mean difference (SE) | 95% CI | p-value |
| Alter gender | Women (n=136) | Ref | | |
|  | Men (n=168) | -0.74 (1.21) | -3.13-1.66 | 0.545 |
| Alter gender x ego gender interaction | Woman x woman (n=84) | Ref | | |
|  | Woman x man (n=52) | 2.64 (1.70) | -0.73-6.01 | 0.124 |
|  | Man x woman (n=119) | -0.29 (1.56) | -3.40-2.81 | 0.853 |
|  | Man x man (n=49) | 1.65 (2.08) | -2.47-5.77 | 0.430 |
| Alter age | Younger than ego (n=271) | Ref | | |
|  | Same age or older than ego (n=33) | 4.16 (1.58) | 1.03-7.29 | 0.010 |
| Relation type | Child (n=195) | Ref | | |
|  | Partner (n=52) | 6.75 (1.12) | 4.46-8.94 | <0.001 |
|  | Other family (n=37) | -3.29 (2.38) | -8.01-1.43 | 0.170 |
|  | Other (n=20) | -6.45 (3.50) | -13.39-0.49 | 0.068 |
| Alter resides in same household | No (n=134) | Ref | | |
|  | Yes (n=170) | 14.66 (1.01) | 12.65-16.67 | <0.001 |
